# Comparison of the Physicochemical Properties, Microbial Communities, and Hydrocarbon Composition of Honeys Produced by Different *Apis* Species

**DOI:** 10.3390/foods13233753

**Published:** 2024-11-23

**Authors:** Guozhi Zhang, Yao Liu, Yaling Luo, Cuiping Zhang, Shanshan Li, Huoqing Zheng, Xiasen Jiang, Fuliang Hu

**Affiliations:** 1 College of Animal Sciences, Zhejiang University, Hangzhou 310058, China; zhangguozhi@zju.edu.cn (G.Z.); lyao0515@163.com (Y.L.); lgzcplyx@zju.edu.cn (C.Z.); lishanshan@zju.edu.cn (S.L.); hqzheng@zju.edu.cn (H.Z.); 2 Engineering Technology Research Center of Anti-Aging Chinese Herbal Medicine of Anhui Province, Biology and Food Engineering School, Fuyang Normal University, Fuyang 236000, China; lyl1358211941@163.com

**Keywords:** *Apis mellifera* honey, *Apis florea* honey, physicochemical properties, bacterial community

## Abstract

The chemical composition and quality of honey are influenced by its botanical, geographic, and entomological origins, as well as climatic conditions. In this study, the physicochemical characteristics, microbial communities, and hydrocarbon compounds of honey produced by *Apis mellifera*, *Apis cerana*, *Apis laboriosa*, *Apis dorsata*, and *Apis florea* were elucidated. The physicochemical profile of the honey exhibited significant differences across species, including moisture content (18.27–23.66%), fructose (33.79–38.70%), maltose (1.10–1.93%), electrical conductivity (0.37–0.74 mS/cm), pH (3.36–3.72), diastase activity (4.50–29.97 diastase number), and color (37.90–102.47 mm). Microbial analysis revealed a significant abundance of lactic acid bacteria, particularly the *Apilactobacillus* genus in *A. laboriosa* honey and the *Lactobacillus* in *A. florea* honey, indicating significant probiotic potential. Chemometric methods, principal component analysis, hierarchical cluster analysis, and orthogonal partial least squares discriminant analysis (OPLS-DA) were used to classify the honey samples based on the 12 beeswax-derived hydrocarbons. The OPLS-DA model demonstrated 100% accuracy in predicting the entomological origin of honey, indicating that specific hydrocarbons are reliable markers for honey classification.

## 1. Introduction

Honey is not only a natural sweetener but also a functional food valued for its biological activity and nutritional properties. Its composition is influenced by multiple factors, including botanical origin, geographic location, honey bee species, and climatic conditions [1]. Among these factors, honey bee species play a critical role as they affect foraging behavior, honey processing methods, and, ultimately, the characteristics of honey [2,3,4]. In China, the rich floral resources support various honey bee species, including domesticated types like *Apis mellifera* and *Apis cerana*, and wild species like *Apis laboriosa*, *Apis dorsata*, and *Apis florea*. This diversity of honey bee species contributes to the substantial variations in the physicochemical properties, chemical composition, and antimicrobial and antioxidant activities of the honey they produce [5,6,7,8,9,10]. For instance, studies have demonstrated that *A. mellifera* honey (AMH) contains a lower moisture content compared to *A. dorsata* honey (ADH) [7] and *A. florea* honey (AFH) [2]. Furthermore, ADH exhibits higher radical scavenging activity and total phenolic content than AMH [8,11].

The microbial composition of honey is an important determinant of its quality [12,13,14]. Bacteria from the *Pseudomonas, Bacillus*, *Paenibacillus*, *Bifidobacterium*, and *Lactobacillus* genera have been detected in honey [15]. These microbes exhibit probiotic properties, aiding in the absorption of essential nutrients and improving defense against harmful pathogens. Identifying the microbial communities in honey could provide insights into its biological activity and contribute to quality control measures.

Furthermore, beeswax is a natural substance produced by honey bees and is mainly composed of hydrocarbons, fatty acids, and esters [16]. The composition of beeswax secreted by different honey bee species differs significantly [17]. During honey harvesting, beeswax can mix with honey, introducing species-specific compounds that have been used to trace the entomological origin of honey [18,19,20]. Recent studies have identified species-specific hydrocarbons such as 17-pentatriacontene and hentriacontane, which are unique to *A. cerana* honey (ACH) and AMH, respectively [19]. However, the current standards for honey quality, such as those set by the Codex Alimentarius [21], are primarily based on AMH and may be inapplicable to honey from other species. Therefore, this study aimed to comprehensively evaluate the physicochemical properties, microbial communities, and hydrocarbon profiles of honey produced by different honey bee species in China. By identifying key differences across species, this study aimed to improve the understanding of honey diversity and provide a scientific foundation for the quality control and traceability of honey based on its entomological origin.

## 2. Materials and Methods

### 2.1. Chemicals

Methanol and acetonitrile were acquired from Merck (Darmstadt, Germany). Sodium chloride, potassium iodide, acetic acid, anhydrous sodium carbonate, and petroleum ether (60–90 °C) were obtained from Sinopharm Chemical Reagent Co., Ltd. (Shanghai, China). The C7–C40 saturated alkane standard and 17-pentatriacontene were acquired from Sigma-Aldrich (Shanghai, China) Trading Co., Ltd. (Shanghai, China).

### 2.2. Samples

Due to the limited quantity of some honey samples and different experimental objectives, the samples used for physicochemical measurements and beeswax composition analysis were partially different.

To analyze physicochemical parameters, 93 honey samples were collected from five honey bee species between 2020 and 2022. These included 20 AMH, 23 ACH, 19 *A. laboriosa* honey (ALH), 21 ADH, and 10 AFH samples (Supplementary Table S1).

To analyze beeswax composition in honey, 106 honey samples were collected between 2014 and 2022, including 25 AMH, 24 ACH, 18 ALH, 20 ADH, and 19 AFH samples (Supplementary Table S2).

Six *A. mellifera* beeswax (AMW) and five *A. cerana* beeswax (ACW) samples were obtained from the experimental apiary of Zhejiang University. *A. laboriosa* beeswax (ALW) samples were obtained from Honghe Hani and Yi Autonomous Prefecture (*n* = 3), Lincang (*n* = 2), and Dehong Dai and Jingpo Autonomous Prefecture (*n* = 2). Six *A. dorsata* beeswax (ADW) and five *A. florea* beeswax (AFW) samples were collected from Xishuangbanna Dai Autonomous Prefecture.

All equipment used for collecting honey and beeswax samples was sterilized, and researchers wore protective gear to avoid contamination. Samples were collected in a clean environment, sealed in sterile containers, and stored at 4 °C until analysis.

### 2.3. Physicochemical Analysis

The moisture content was determined using an Abbe refractometer (NAR-2T, Atago Co., Ltd., Tokyo, Japan) [22]. The pH, electrical conductivity (EC), and diastase activity of the honey were measured according to the standard methods described by de Alnneida-Muradian et al. [23]. Color grades (0–150 mm) were measured by inserting the cuvette into a color photometer (HI 96785; Hanna Instruments, Nusfalau, Romania). The sugar profile of honey, including fructose, glucose, sucrose, and maltose, was determined using high-performance liquid chromatography (HPLC; LC-20ADXR, Shimadzu, Kyoto, Japan), equipped with a refractive index detector [24]. In total, 10 μL of honey aqueous solution (0.02 g/mL) was analyzed using a Sepax HP-Amino column (4.6 mm × 250 mm, 5 μm) at 40 °C with a mobile phase of acetonitrile/water (70:30, *v*/*v*), and at a flow rate of 1.0 mL/min. The hydroxymethylfurfural (HMF) content in honey was determined according to the National Standard of China (GB/T 18932.18-2003) [25]. Then, 10 g of honey was mixed with 10 mL of methanol, and then diluted to 100 mL with ultrapure water in a 100 mL volumetric flask. Next, 10 μL of samples were separated using the Shimadzu LC-20ADXR HPLC system, equipped with a UV detector and a Sepax HP-C18 column (4.6 mm × 250 mm, 5 μm). Methanol–water (10:90, *v*/*v*) was used as mobile phase at a flow rate of 1.0 mL/min. The column temperature was maintained at 30 °C, and the detection wavelength was set at 285 nm.

### 2.4. DNA Extraction and 16S rRNA Gene Sequencing

A total of 12 g of honey was dissolved in 25 mL of phosphate-buffered saline (PBS; pH 7.4) and incubated for 20 min at 40 °C. The sample was then centrifuged at 10,000 rpm for 15 min, and the supernatant was discarded. The pellet was resuspended in 10 mL of PBS, and centrifugation was repeated. DNA was extracted from the pellet using the Cetyltrimethylammonium Bromide method. The honey DNA was amplified using universal primers targeting the V3-V4 region of the 16S rRNA gene (341F: 5′-CCTAYGGGRBGCASCAG-3′, 806R: 5′-GGACTACNNGGGTATCTAAT-3′) [26]. Sequencing was performed by Novogene Co., Ltd. (Tianjin, China) using the NovaSeq 6000 platform.

Each sample’s reads were first merged using FLASH software (version 1.2.11) [27], then filtered using fastp software (version 0.23.1) [28]. The filtered data were aligned to the Silva database (https://www.arb-silva.de/, accessed on 4 July 2024) to remove chimeric sequences, producing the final dataset [29]. Denoising was performed using QIIME2’s DADA2 module, resulting in Amplicon Sequence Variants (ASVs) and their feature table [30]. ASVs were annotated using the classify-sklearn algorithm in QIIME2 [31] with a pre-trained Naive Bayes classifier (Silva 138.1), and the chloroplast and mitochondrial sequences were removed. Rarefaction curves were plotted for each species. The top 10 bacterial phyla and genera were visualized using bar charts in Perl software (version 5.26.2; https://www.perl.org/, accessed on 4 July 2024), and linear discriminant analysis effect size (LEfSe) software (version 1.1.01) [32] was used for differential analysis between sample groups.

### 2.5. Determination of Beeswax Composition in Honey Samples

#### 2.5.1. Sample Preparation

The honey samples were pretreated according to the method described by Zhang et al. [19] with minor modifications. Approximately 20 g of honey was dissolved in 100 mL of distilled water and centrifuged at 5000 rpm for 10 min to remove pollen. The supernatant was vacuum-filtered using slow-filter paper. The filter paper was then immersed in 15 mL of petroleum ether (60–90 °C) and subjected to ultrasound treatment for 20 min. The resulting solution was evaporated using a nitrogen sample concentrator (MD 200-1, Hangzhou Allsheng Instruments Co., Ltd., Hangzhou, Zhejiang, China) and redissolved in 2 mL of petroleum ether (60–90 °C).

Beeswax was ultrasonically cleaned twice for 10 min using distilled water. Then, 20 mg of beeswax was dissolved in petroleum ether (60–90 °C) to prepare a 4 mg/mL solution. The petroleum ether extracts of honey and beeswax were filtered through a 0.22 μm membrane filter before use.

#### 2.5.2. High-Temperature Gas Chromatography

The analysis was conducted using the method described by Zhang et al. [19] with appropriate modifications. The analyses were performed using an Agilent 7890B gas chromatograph coupled with an Agilent 5977B mass spectrometer (Agilent, Santa Clara, CA, USA) for gas chromatography–mass spectrometry (GC-MS), and an Agilent 7890A gas chromatograph equipped with a flame ionization detector (Agilent, Santa Clara, CA, USA) for gas chromatography–flame ionization detection (GC-FID). Both GC analyses were carried out under the same conditions. The HP-5 capillary column (30 m × 0.32 mm internal diameter, 0.25 μm film thickness) was used as the chromatographic column. Helium (99.999% purity) was used as a carrier gas at a constant flow rate of 0.8 mL/min. Each time, a 1 μL sample was injected with a split ratio of 5:1. The injection port temperature was set at 250 °C. The oven temperature program was as follows: the initial temperature was held at 40 °C for 3 min, then increased at a rate of 20 °C/min to 280 °C and maintained for 20 min. The analysis was completed in 35 min.

For GC-MS analysis, the scanning range was 30–550 amu, and the Agilent Mass Hunter software (version B.07.00) was used as the search engine. Qualitative analysis of the compounds was performed by comparing with the National Institute of Standards and Technology (NIST 17) mass spectral database (with a matching degree > 90%) and validated using reference standard substances.

### 2.6. Statistical Analysis

The physicochemical properties of honey are expressed as the mean ± standard deviation and [minimum-maximum]. Statistical analyses were performed using the statistical package for the social sciences software (version 22.0; SPSS, Chicago, IL, USA). The Shapiro–Wilk test was used to assess the normality of the data. Parametric tests (for instance, one-way analysis of variance) were conducted for data with a normal distribution (*p* > 0.05). Tukey’s post hoc test was conducted when Levene’s test for equality of variances was significant (*p* > 0.05), indicating homogeneity of variance. If Levene’s test was significant (*p* < 0.05), it indicated the heterogeneity of variances. Dunnett’s T3 test was used for multiple comparisons. If the data did not meet the normality assumption, non-parametric tests (Kruskal–Wallis test) were used to analyze differences. A *p* < 0.05 indicated significant differences. Soft independent modeling of class analogy software (version 14.1; MKS Umetrics, Malmö, Sweden) was used for chemometric analyses on the hydrocarbon components of honey from different honey bee species, including principal component analysis (PCA), hierarchical cluster analysis (HCA), and orthogonal partial least squares discriminant analysis (OPLS-DA).

## 3. Results and Discussion

### 3.1. Physicochemical Properties of Honey from Different Honey Bee Species

In this study, the physicochemical parameters of AMH, ACH, ALH, ADH, and AFH in China were determined (Table 1). Moisture content is one of the key parameters for determining the maturity and stability of honey. If the moisture content is too high, it becomes a key factor in honey fermentation and changes the sensory characteristics [33,34]. The European Commission and the Codex Alimentarius Commission have set a maximum moisture content of 20% for honey [21,35]. In all honey samples, the moisture content of AMH was 18.27 ± 1.03%, significantly lower than that of ACH (20.84 ± 1.64%), ALH (23.29 ± 1.07%), ADH (23.66 ± 1.25%), and AFH (23.26 ± 0.56%) (*p* < 0.05). This is consistent with Berenbaum and Calla [36], who also reported that the moisture content of AMH is lower than that of honey obtained from other honey bee species. The moisture contents of AMH and ADH in this study matched the results of Wu et al. [7]. The moisture content of honey is influenced by various factors, including weather, plants, and honey bee species [11,37,38]. Given that the moisture contents of ACH, ALH, ADH, and AFH exceed 20%, there may be a need to establish different standards for these varieties.

The primary monosaccharides in honey include fructose (32–44%) and glucose (23–38%) [39]. AMH exhibited the highest fructose content (38.70 ± 1.92%), while ADH exhibited the lowest (33.79 ± 3.07%). However, there were no significant differences in the glucose content of the honey samples. The reducing sugar contents (fructose and glucose) exhibited a similar pattern, with AMH exhibiting the highest level at 70.72 ± 4.49% and ADH the lowest at 63.38 ± 4.83%. In this study, all honey samples met the required minimum of 60%, except for the six ADH samples [21,35].

The sucrose content in ALH was 3.79 ± 1.32%, significantly higher than that in honey from other species (*p* < 0.05). All honey samples analyzed exhibited sucrose levels below 5%, except for five samples in the ALH group. The maltose content in ADH (1.93 ± 0.56%) was significantly higher than that in AMH (1.10 ± 0.36%) and ACH (1.40 ± 0.74%). Additionally, the maltose contents in ALH (1.65 ± 0.41%) and AFH (1.67 ± 0.40%) were significantly higher than that in AMH (*p* < 0.05). The enzymatic reactions involved in the conversion of nectar into honey, as well as non-enzymatic reactions during honey storage, can influence the sugar composition of honey. Due to these factors, different honey bee species may produce honey with unique sugar profiles. For instance, studies have discovered that trehalose is a characteristic sugar in stingless bee honey [40,41]. Therefore, besides the common sugars, further research is required to identify other sugars in honey.

The EC of honey is related to its mineral, organic acid, and protein contents. In this study, the EC of ALH was 0.74 ± 0.41 mS/cm, significantly higher than that of AFH (0.50 ± 0.83 mS/cm), AMH (0.43 ± 0.21 mS/cm), and ACH (0.37 ± 0.18 mS/cm) (*p* < 0.05). However, there was no significant difference between ALH and ADH (0.53 ± 0.16 mS/cm) (*p* > 0.05). Except for six samples from the ALH group and one from the ADH group, the EC of all honey samples was below the specified limit of 0.8 mS/cm. This may indicate that they were honeydew honey, as the EC of honeydew honey should not be less than 0.8 mS/cm [21,35]. Additionally, Yang et al. [6] discovered that few ALH, ADH, and AFH samples exceeded the limit. Wu et al. [7] indicated that the EC of ADH (0.55 ± 0.00 mS/cm) was significantly higher than that of AMH (0.24 ± 0.00 mS/cm) and ACH (0.40 ± 0.00 mS/cm). However, in our study, ADH demonstrated no significant difference in EC compared to AMH and ACH, which may be attributed to differences in the honey sample sources.

Although honey standards do not specify a specific pH range, fresh honey typically exhibits a pH below 5.5 [24,42]. The pH values of all analyzed honey samples ranged from 2.77 to 4.10, with only AFH (3.72 ± 0.15) exhibiting a significant difference compared to ACH (3.36 ± 0.14) (*p* < 0.05). Yap et al. also observed similar values for AMH and ADH [8].

Diastase activity is considered an important indicator for assessing the freshness of honey [43]. Relevant standards demonstrate that the diastase activity of honey should not be lower than 8 Schade units, except for certain naturally low-diastase honey types, which exhibit a value of at least 3 Schade units [21,35]. The diastase activity of AMH was 29.97 ± 19.66 DN, significantly higher than that of honey from other honey bee species (*p* < 0.05). This high activity may be related to the significant colony strength of *Apis mellifera* and the longer honey maturation period, both of which likely promote enzyme secretion and accumulation. The diastase activity of ALH and AFH aligned with the data reported by Yang et al. [6]. Diastase activity in honey is influenced by its botanical origin [44]; however, studies demonstrate that honey from the same plant exhibits significantly lower diastase activity in AFH than in AMH [45], indicating that it is also related to honey bee species.

Honey color varies from water white to dark amber [46]. The color of ALH (102.47 ± 36.52 mm, amber) and AFH (79.20 ± 35.46 mm, light amber) was significantly higher than that of AMH (37.90 ± 26.88 mm, extra light amber), ACH (50.83 ± 27.00 mm, light amber), and ADH (48.95 ± 19.97 mm, extra light amber) (*p* < 0.05). The color of honey is primarily influenced by its botanical origin and probably by the storage temperature [47]. Additionally, there are few reports on the colors of ALH, ADH, and AFH.

HMF is a key indicator of honey freshness and heat treatment [37], and its concentration must not exceed 40 mg/kg [21,35]. The HMF content of the honey samples ranged from 0.59 to 13.72 mg/kg, significantly below the specified limit, indicating that the analyzed honey samples were fresh.

### 3.2. Diversity of Bacteriome in Honey Produced by Different Honey Bee Species

Microorganisms in honey can potentially affect their safety and quality [15]. The main sources of microorganisms in honey are the gut microbiota of honey bees, environmental microbes (from soil, air, plants, and flowers) acquired during foraging, and those found inside beehives [48,49]. The DADA2 method was used to cluster sequences with 100% similarity, leading to the identification of 4497 amplicon sequence variants (ASVs). These ASVs were annotated for species in the Silva 138.1 database, resulting in the identification of 34 phyla, 74 classes, 174 orders, 289 families, and 648 genera. The number of species annotated at each taxonomic level varied among the different types of honey (Supplementary Table S3).

**Figure 1:**
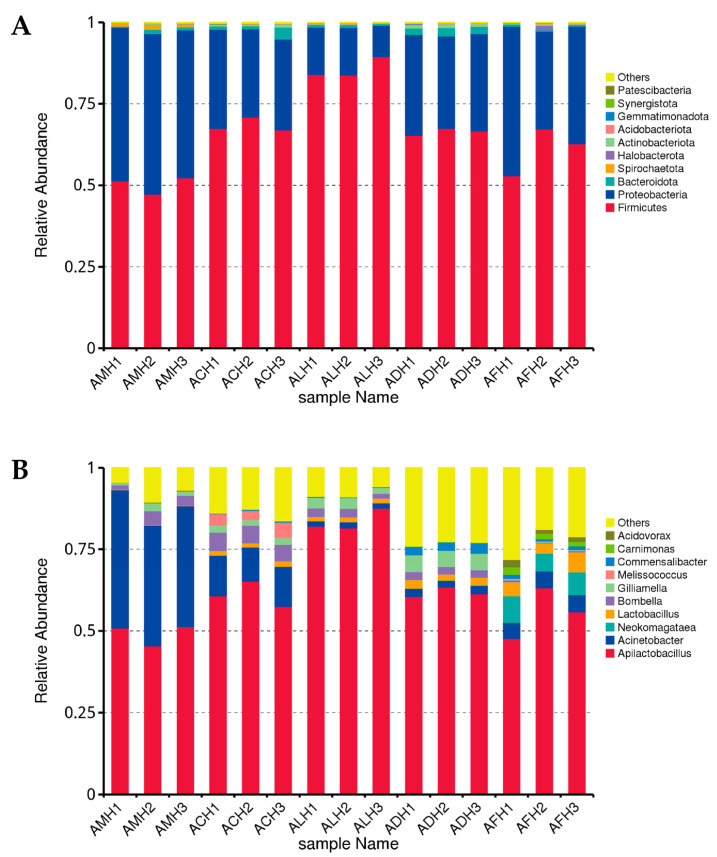
The relative abundance of bacterial communities in honey from different honey bee species at the (**A**) phylum and (**B**) genus levels. The *x*-axis represents the sample names, and the *y*-axis indicates the relative abundance. Others represent the sum of the relative abundances of all other phyla (or genera) not included in the top 10 phyla (or genera). AMH: *A. mellifera* honey; ACH: *A. cerana* honey; ALH: *A. laboriosa* honey; ADH: *A. dorsata* honey; AFH: *A. florea* honey.

Based on the results of the ASV annotation, we selected the top 10 dominant species at the phylum and genus levels for each sample and created abundance histograms (Figure 1). At the phylum level, all honey samples were primarily composed of Firmicutes, with the lowest relative abundance in the AMH samples (50.29%) and the highest in the ALH samples (85.75%). Other phyla included Proteobacteria, Bacteroidota, and Actinobacteriota, with relative abundances of 47.23%, 0.88%, and 0.22% in AMH samples; 28.43%, 1.87%, and 0.66% in ACH samples,12.90%, 0.74%, and 0.23% in ALH samples; 29.70%, 2.23%, and 1.01% in ADH samples; and 37.31%, 0.49%, and 0.35% in AFH samples (Figure 1A). The relative abundance of Proteobacteria in ALH samples was significantly lower than that in AMH samples (*p* < 0.05), while there were no significant differences in the abundance of Bacteroidota across the five types of honey. Yap et al. [8] discovered that the top three phyla in AMH were Firmicutes, Proteobacteria, and Actinobacteriota, while the top three in ADH were Actinobacteriota, Proteobacteria, and Bacteroidota. The differences observed in this study may be primarily attributed to variations in the sample origin and quality. Firmicutes, Proteobacteria, and Actinobacteriota are facultative anaerobes that can decompose and ferment carbohydrates [50]. Consequently, these three phyla play a crucial role in the honey maturation process, elucidating their status as the predominant microbial communities in honey [8].

The genus *Apilactobacillus* was predominant in AMH, ACH, ALH, ADH, and AFH samples, with relative abundances of 49.21%, 61.16%, 83.75%, 61.77%, and 55.59%, respectively. Notably, the relative abundance of this genus was significantly higher in ALH than in AMH samples (*p* < 0.05; Figure 1B). *Apilactobacillus* is a fructophilic lactic acid bacterium, thriving in fructose-rich environments such as the gut of honey bees. Furthermore, the relative abundance of *the Lactobacillus* genus in AMH, ACH, ALH, ADH, and AFH was 0.14%, 1.46%, 1.42%, 2.30%, and 4.54%, respectively. These lactic acid bacteria, which are major components of honey bee gut microbiota [51], possess probiotic properties essential for bee health and contribute to the antibacterial properties of honey [52,53]. ALH exhibited the highest abundance of *Apilactobacillus*, while *Lactobacillus* was prevalent in AFH. This suggests that ALH and AFH exhibit significant probiotic potential. In the AMH samples, the relative abundance of *Lactobacillus* was the lowest, which may be attributed to the moisture content of the honey. When the moisture content of honey is around 18%, the growth and survival of *Lactobacillus* could be inhibited [54,55]. The relative abundance of the *Acinetobacter* genus was 38.79% in AMH, 11.70% in ACH, 1.69% in ALH, 2.35% in ADH, and 5.08% in AFH. Wu et al. [9] discovered that *Acinetobacter* was prevalent in ACH, ADH, and AMH, with relative abundances of 27.29%, 20.44%, and 24.18%, respectively. This difference may be attributed to variations in honey sources and sequencing techniques. Research has demonstrated that the DADA2 method is more sensitive and specific than traditional operational taxonomic unit (OTU) methods [56].

**Figure 2:**
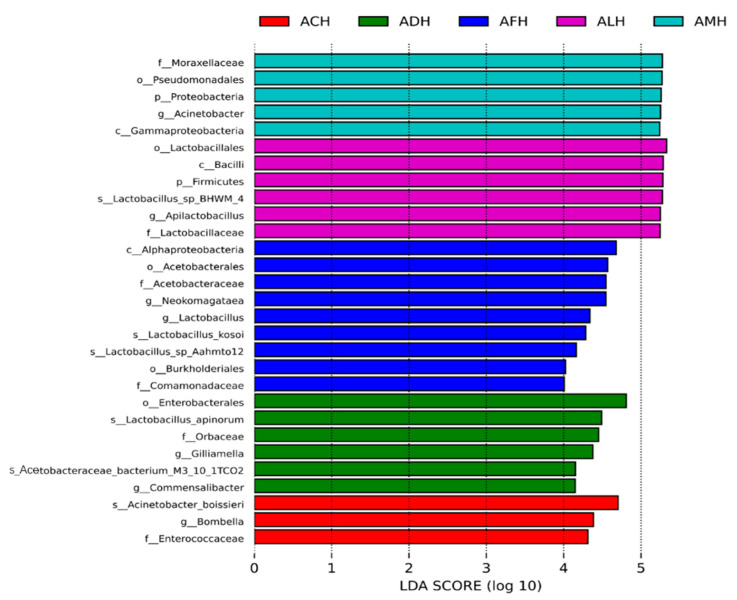
LEfSe analysis of bacterial communities in honey from different honey bee species. p: phylum; c: class; o: order; f: family; g: genus; s: species; AMH: *A. mellifera* honey; ACH: *A. cerana* honey; ALH: *A. laboriosa* honey; ADH: *A. dorsata* honey; AFH: *A. florea* honey.

LEfSe is an analytical tool specifically designed to identify and explain statistically significant differences in high-dimensional biological data, particularly biomarkers. In this study, species with a linear discriminant analysis score > 4 were selected to create bar charts (Figure 2). *Acinetobacter*, *Bombella*, and *Apilactobacillus* genera were remarkably enriched in the AMH, ACH, and ALH samples, respectively. The *Gilliamella* and *Commensalibacter* genera were significantly more abundant in ADH samples, while *Neokomagataea* and *Lactobacillus* genera showed higher abundance in AFH samples. Additionally, bacterial communities in honey from different honey bee species exhibited significant differences at each taxonomic level. These variations in microbial communities likely reflect differences in the microecological environments related to honey production across different honey bee species.

### 3.3. Analysis of Beeswax Composition in Honey Samples

**Figure 3:**
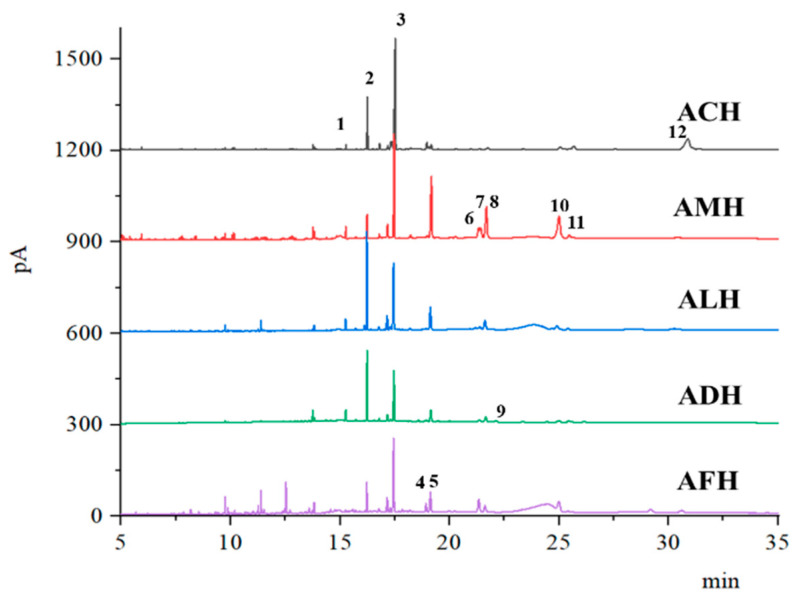
Gas chromatography of petroleum ether extracts from honey of different honey bee species. AMH: *A. mellifera* honey; ACH: *A. cerana* honey; ALH: *A. laboriosa* honey; ADH: *A. dorsata* honey; AFH: *A. florea* honey.

GC-MS analysis revealed that the chromatographic peaks of petroleum ether extracts from honey of the same species were similar, while those from different species exhibited significant differences (Figure 3). Further analysis revealed that the distinctive peaks were primarily due to variations in the beeswax composition (Supplementary Figure S1). Since beeswax is secreted directly by the wax glands of honey bees, variations in honey bee genotypes may directly contribute to differences in beeswax composition [17]. In our study, we identified 12 hydrocarbon compounds derived from beeswax in honey samples (Table 2). The relative abundance (%) of the identified hydrocarbon compounds was determined using the peak area of the most abundant hydrocarbon heptacosane. Heptacosane exhibited the highest relative content in honey samples from most honey bee species, while pentacosane exhibited the highest relative content in the ALH group. Additionally, the isomers of hentriacontene, 17-tritriacontene, and 15-methylnonacosane were detected only in AMH, ACH, and ADH, respectively, indicating that these compounds could be characteristic markers for honey of these specific species. A previous study observed that 17-pentatriacontene and hentriacontane act as species-specific markers for ACH and AMH, respectively [19]. However, we also detected hentriacontane in ALH, ADH, and AFH. Although hentriacontane can serve as a distinguishing compound between AMH and ACH, its specificity is unreliable when considering honey from other species.

#### 3.3.1. PCA

**Figure 4:**
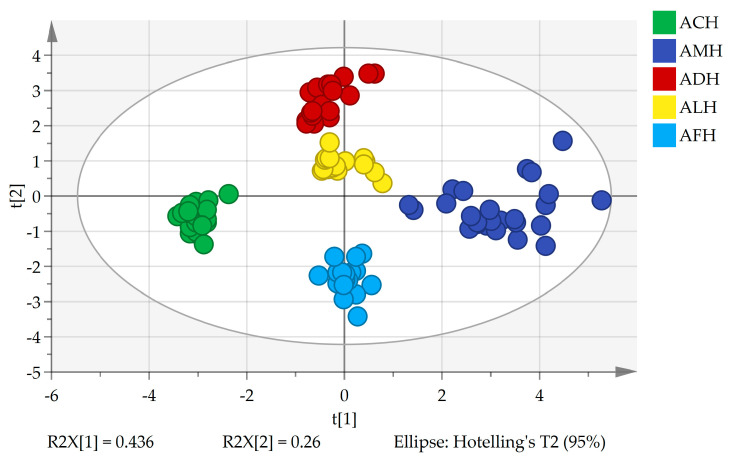
PCA score plot of 12 hydrocarbon compounds from different honey samples. AMH: *A. mellifera* honey; ACH: *A. cerana* honey; ALH: *A. laboriosa* honey; ADH: *A. dorsata* honey; AFH: *A. florea* honey.

PCA is a well-known unsupervised model widely used to explain the variance of variables in large datasets [57]. The PCA score plot for the first two principal components, PC1 and PC2, which explained 69.6% of the total variance, is depicted in Figure 4. PC1 accounted for 43.6%, and PC2 accounted for 26.0%. Honey samples from the same species were clustered together, with PC1 distinguishing ACH from AMH and PC2 differentiating ADH, ALH, and AFH. The distribution of the five honey types in the principal component space was relatively dispersed and distinct, indicating significant differences in their hydrocarbon composition.

#### 3.3.2. HCA

**Figure 5:**
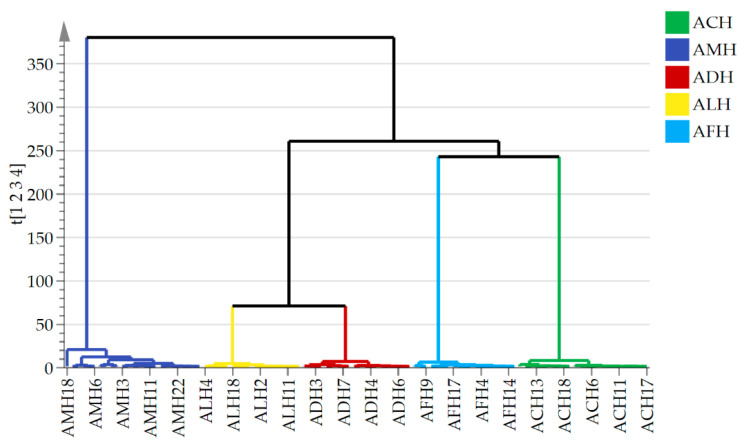
HCA of 12 hydrocarbon compounds from different honey samples. AMH: *A. mellifera* honey; ACH: *A. cerana* honey; ALH: *A. laboriosa* honey; ADH: *A. dorsata* honey; AFH: *A. florea* honey.

HCA is a commonly used clustering analysis method designed to group samples within a dataset based on their similarities. Pre-specification of the number of clusters is not required for this method; instead, it gradually aggregates samples into different clusters based on the distance or similarity measures within the data [58]. The HCA results indicated that all honey samples could be divided into five groups (Figure 5). ACH and AFH were closely related and clustered, while ALH and ADH exhibited similar hydrocarbon compositions, forming another cluster. Contrarily, AMH was the most distantly related to the other samples, forming a separate cluster.

#### 3.3.3. OPLS-DA

**Figure 6:**
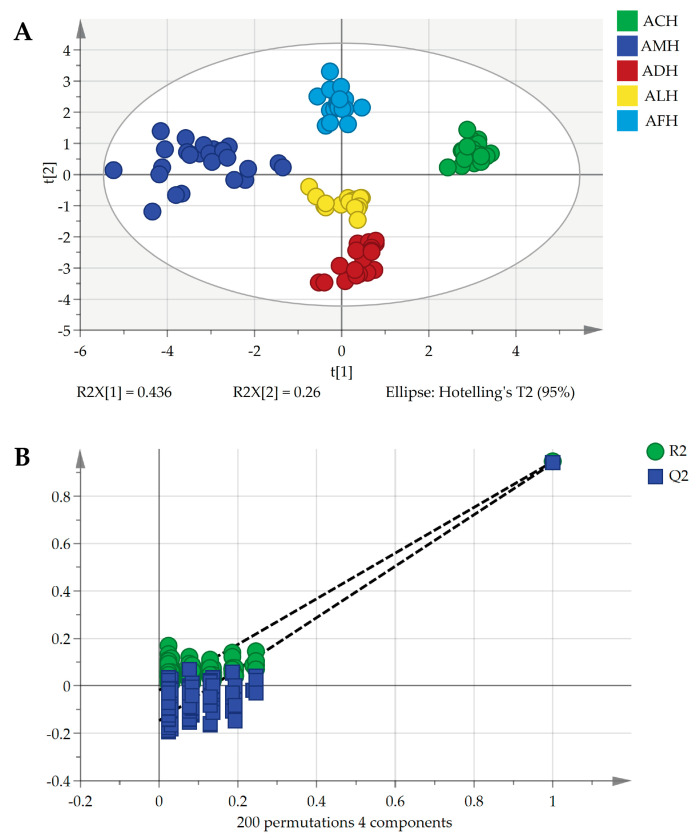
OPLS-DA score plot of different honey samples (**A**,**B**) permutation test of the OPLS-DA model with 200 random permutations. AMH: *A. mellifera* honey; ACH: *A. cerana* honey; ALH: *A. laboriosa* honey; ADH: *A. dorsata* honey; AFH: *A. florea* honey.

OPLS-DA is a supervised chemometric method that improves the performance and interpretability of classification models by introducing orthogonal components [59]. The score plot of the OPLS-DA model for honey from different honey bee species, established based on 12 beeswax-derived hydrocarbon compounds, is illustrated in Figure 6A. The plot revealed that honey samples were clustered according to species; honey from different species was relatively dispersed, consistent with the results of PCA and HCA. The model evaluation parameters R^2^Y (cum) = 0.936, Q^2^ (cum) = 0.930, and R^2^Y − Q^2^ = 0.006 indicate that the model exhibits excellent fit and predictive capability. It is generally accepted that substances with a variable importance in projection (VIP) value > 1 significantly contribute to the model interpretation. In this model, hydrocarbon compounds with VIP values > 1 included 15-methylnonacosane (VIP = 1.18), pentacosane (VIP = 1.18), nonacosene (VIP = 1.05), and hentriacontene (VIP = 1.02).

A permutation test (*n* = 200) was conducted to evaluate the risk of overfitting in the model further. The result yielded R^2^ = −0.0188 and Q^2^ = −0.147 from 200 permutation tests, with Q^2^ representing a negative intercept on the *Y*-axis, indicating that the model was not prone to overfitting and demonstrated strong stability and predictive performance (Figure 6B).

Furthermore, the established model was used to treat the working set as the prediction set, yielding a misclassification table. The misclassification table indicates that the model achieved a prediction accuracy of 100%, with all samples accurately classified and none misclassified into other categories (Table 3). These results confirm the high accuracy and reliability of the model.

## 4. Conclusions

The physicochemical parameters and bacterial profiles of honey produced by the five honey bee species in China varied significantly. AMH had the lowest moisture content (18.27 ± 1.03%), the highest fructose concentration (38.70 ± 1.92%), and the highest diastase activity (29.97 ± 19.66 DN), while honey from other species had moisture levels above 20%. These differences highlight the need for species-specific honey standards. Furthermore, the high abundance of lactic acid bacteria in ALH and AFH suggests probiotic potential. However, since honey composition is influenced by factors such as plant source, climate, and geography, these characteristics alone cannot definitively identify the insect source of honey. They can only serve as supplementary indicators. Based on the 12 beeswax-derived hydrocarbon compounds, PCA, HCA, and OPLS-DA were used to differentiate honey from the five honey bee species. The developed OPLS-DA model accurately predicted the entomological origin of honey with a prediction accuracy of 100%. These findings not only provide a reliable scientific basis for assessing honey quality but also offer a novel approach for accurately identifying the entomological origin of honey.

## Figures and Tables

**Table 1:** Physicochemical properties of honey samples from different honey bee species.

Parameters	AMH	ACH	ALH	ADH	AFH
Moisture (%)	18.27 ± 1.03 c [16.57–19.97]	20.84 ± 1.64 b [17.86–23.48]	23.29 ± 1.07 a [21.14–25.13]	23.66 ± 1.25 a [20.98–26.26]	23.26 ± 0.56 a [22.31–23.84]
Fructose (%)	38.70 ± 1.92 a [35.20–43.86]	35.56 ± 2.29 bc [31.39–39.78]	35.08 ± 0.68 bc [33.45–36.06]	33.79 ± 3.07 c [27.13–38.44]	36.26 ± 0.92 b [34.61–37.68]
Glucose (%)	32.02 ± 4.16 a [24.41–37.16]	30.28 ± 3.50 a [23.44–36.76]	30.24 ± 1.24 a [27.53–31.83]	29.59 ± 1.97 a [25.68–33.08]	31.73 ± 1.90 a [27.73–33.97]
Reducing sugar (%)	70.72 ± 4.49 a [62.32–76.86]	65.84 ± 4.20 bc [60.15–74.16]	65.32 ± 1.55 bc [61.82–67.89]	63.38 ± 4.83 c [53.77–71.52]	67.99 ± 2.08 ab [64.63–71.06]
Sucrose (%)	2.46 ± 1.25 b [0.64–4.62]	2.54 ± 1.15 b [0.34–4.66]	3.79 ± 1.32 a [1.84–5.59]	2.65 ± 0.98 b [1.24–4.38]	2.45 ± 0.42 b [1.78–3.18]
Maltose (%)	1.10 ± 0.36 c [0.38–1.95]	1.40 ± 0.74 bc [0.43–2.82]	1.65 ± 0.41 ab [0.89–2.29]	1.93 ± 0.56 a [0.89–2.93]	1.67 ± 0.40 ab [1.00–2.21]
EC (mS/cm)	0.43 ± 0.21 b [0.15–0.75]	0.37 ± 0.18 b [0.13–0.74]	0.74 ± 0.41 a [0.30–1.45]	0.53 ± 0.16 ab [0.16–0.83]	0.50 ± 0.83 b [0.38–0.65]
pH	3.52 ± 0.27 ab [3.10–4.10]	3.36 ± 0.14 b [3.11–3.76]	3.61 ± 0.56 ab [2.77–4.30]	3.61 ± 0.21 ab [3.34–4.02]	3.72 ± 0.15 a [3.54–3.95]
Diastase activity (DN)	29.97 ± 19.66 a [10.60–66.67]	9.98 ± 4.61 b [5.60–20.69]	4.54 ± 0.23 b [4.11–4.84]	4.50 ± 0.41 b [4.00–5.42]	13.79 ± 5.63 b [7.69–25.00]
Color (mm)	37.90 ± 26.88 b [3.00–86.00]	50.83 ± 27.00 b [6.00–101.00]	102.47 ± 36.52 a [22.00–150.00]	48.95 ± 19.97 b [13.00–82.00]	79.20 ± 35.46 a [28.00–150.00]
HMF (mg/kg)	2.07 ± 1.39 b [0.65–5.90]	5.70 ± 4.71 a [0.75–13.72]	2.04 ± 1.00 b [0.71–3.75]	1.11 ± 0.29 b [0.59–1.75]	1.32 ± 0.46 b [0.68–2.03]

Data are expressed as the mean ± standard deviation and [minimum-maximum]. Different lower-case letters in the same row correspond to significant differences (*p* < 0.05). EC: electrical conductivity; DN: diastase number; AMH: *A. mellifera* honey; ACH: *A. cerana* honey; ALH: *A. laboriosa* honey; ADH: *A. dorsata* honey; AFH: *A. florea* honey.

**Table 2:** Chemical composition of petroleum ether extracts from honey of different honey bee species.

No.	Compound Name	Abbreviation	RT (min)	Amount (%)				
				AMH	ACH	ALH	ADH	AFH
1	Tricosane	HC 23:0	15.28	9.53	4.87	8.89	10.04	2.05
2	Pentacosane	HC 25:0	16.25	22.04	35.28	109.92	95.93	23.39
3	Heptacosane	HC 27:0	17.48	100.00	100.00	100.00	100.00	100.00
4	Nonacosene	HC 29:1	18.95	0.00	5.47	3.64	2.90	17.48
5	Nonocosane	HC 29:0	19.18	74.06	5.07	45.86	32.19	42.07
6	Hentriacontene	HC 31:1	21.35	18.74	0.00	9.83	5.88	35.24
7	Hentriacontene isomer	HC 31:1	21.45	16.90	0.00	0.00	0.00	0.00
8	Hentriacontane	HC 31:0	21.69	56.90	0.00	24.20	23.79	20.70
9	15-Methylnonacosane	HC 30:1	22.10	0.00	0.00	0.00	10.01	0.00
10	Tritriacontene	HC 33:1	25.00	64.98	7.45	30.15	11.00	40.36
11	Tritriacontane	HC 33:0	25.43	10.70	0.00	6.73	13.86	5.32
12	17-Pentatriacontene	HC 35:1	30.73	0.00	49.66	0.00	0.00	0.00

RT: retention time; the amount (%) was calculated from the peak area with reference to the peak area of the most abundant hydrocarbon, 27:0; AMH: *A. mellifera* honey; ACH: *A. cerana* honey; ALH: *A. laboriosa* honey; ADH: *A. dorsata* honey; AFH: *A. florea* honey.

**Table 3:** The misclassification table for the OPLS-DA model of different honey samples.

	Number	Accuracy	AMH	ACH	ALH	ADH	AFH	Unclassified
AMH	25	100%	25	0	0	0	0	0
ACH	24	100%	0	24	0	0	0	0
ALH	18	100%	0	0	18	0	0	0
ADH	20	100%	0	0	0	20	0	0
AFH	19	100%	0	0	0	0	19	0
Unclassified	0	100%	0	0	0	0	0	0
Total	106	100%	25	24	18	20	19	0

AMH: *A. mellifera* honey; ACH: *A. cerana* honey; ALH: *A. laboriosa* honey; ADH: *A. dorsata* honey; AFH: *A. florea* honey.

## Data Availability

The original contributions presented in the study are included in the article/Supplementary Material, further inquiries can be directed to the corresponding authors.

## Supplementary Materials

The following supporting information can be downloaded at: https://www.mdpi.com/article/10.3390/foods13233753/s1, Table S1: The information of honey samples in China; Table S2: Information on honey samples from China for hydrocarbon composition analysis; Table S3: Sequencing data of microbial community in honey samples.
