# Supplementary material for: Comparison of the Physicochemical Properties, Microbial Communities, and Hydrocarbon Composition of Honeys Produced by Different Apis Species
Source: Foods. 2024 Nov 23;13(23):3753. doi: 10.3390/foods13233753 (PMC11640217; doi:10.3390/foods13233753)
Supplement: Supplementary file 1 [file foods-13-03753-s001.zip › foods-3305964-supplementary.pdf]

Table S1. The information of honey samples in China

	Botanical source	Harvest Date	Geographical Origin	Number
Apis mellifera honey (AMH)	*Foeniculum vulgare* Mill.	2020. 07	Wuwei, Gansu	2
	Brassica napus L.	2020. 03	Quzhou, Zhejiang	1
	Polyflora	2020. 07	Golmud, Qinghai	2
	Scrophularia ningpoensis Hemsl.	2020. 07	Bozhou, Anhui	2
	Vitex negundo L.	2021. 07	Xingtai, Hebei	3
	Polyflora	2021. 06	Shaoxing, Zhejiang	3
	Polyflora	2022. 05	Hangzhou, Zhejiang	2
	Robinia pseudoacacia L.	2022. 05	Yan'an, Shaanxi	2
	Brassica napus L.	2022. 04	Jinhua, Zhejiang	3
Apis cerana honey (ACH)	*Brassica napus* L.	2020. 03	Quzhou, Zhejiang	2
	Polyflora	2020. 05	Hangzhou, Zhejiang	2
	Rhus chinensis Mill.	2020. 06	Yan'an, Shaanxi	2
	Polyflora	2020. 11	Shennongjia Forestry District, Hubei	2
	Polyflora	2021. 04	Wenzhou, Zhejiang	2
	Triadica cochinchinensis	2021. 05	Shangrao, Jiangxi	1
	Leucosceptrum canum Smith	2021. 05	Dehong, Yunnan	2
	Polyflora	2021. 11	Enshi, Hubei	2
	Eriobotrya japonica Lindl.	2021. 12	Hangzhou, Zhejiang	3
	Polyflora	2022. 03	Hangzhou, Zhejiang	2
	Litchi chinensis Sonn.	2022. 03	Huizhou, Guangdong	2
	Polyflora	2022. 05	Chongzuo, Guangxi	1
Apis laboriosa honey (ALH)	Polyflora	2020. 04	Honghe, Yunnan	4
	Polyflora	2020. 05	Pu'er, Yunnan	1
	Polyflora	2020. 05	Lincang, Yunnan	3
	Polyflora	2021. 04	Xishuangbanna, Yunnan	1
	Polyflora	2021. 05	Dehong, Yunnan	5
	Polyflora	2022. 04	Dehong, Yunnan	5
Apis dorsata honey (ADH)	Polyflora	2020. 06	Xishuangbanna, Yunnan	6
	Polyflora	2021. 04	Xishuangbanna, Yunnan	4
	Polyflora	2021. 06	Xishuangbanna, Yunnan	4
	Polyflora	2022. 04	Xishuangbanna, Yunnan	5
	Polyflora	2022. 05	Chongzuo, Guangxi	1
	Polyflora	2022. 05	Baise, Guangxi	1
Apis florea honey (AFH)	Polyflora	2021. 04	Xishuangbanna, Yunnan	3
	Polyflora	2021. 06	Xishuangbanna, Yunnan	4
	Polyflora	2022. 04	Chongzuo, Guangxi	2
	Polyflora	2022. 06	Honghe, Yunnan	1

Table S2. Information on honey samples from China for hydrocarbon composition analysis

	Botanical source	Harvest Date	Geographical Origin	Number
Apis mellifera honey (AMH)	*Sapindus saponaria* L.	2014. 06	Jinhua, Zhejiang	2
	Polyflora	2015. 04	Yan'an, Shaanxi	2
	Ilex chinensis Sims	2015. 06	Jinhua, Zhejiang	1
	Tilia amurensis Rupr.	2016. 07	Baishan, Jilin	2
	Glochidion puberum (L.) Hutch.	2017. 07	Ningbo, Zhejiang	1
	Litchi chinensis Sonn.	2018. 04	Shenzhen, Guangdong	2
	Foeniculum vulgare Mill.	2020. 07	Wuwei, Gansu	3
	Brassica napus L.	2020. 03	Quzhou, Zhejiang	3
	Ziziphus jujuba Mill.	2020. 05	Yan'an, Shaanxi	2
	Scrophularia ningpoensis Hemsl.	2020. 07	Bozhou, Anhui	2
	Polyflora	2021. 11	Shaoxing, Zhejiang	2
	Robinia pseudoacacia L.	2022. 05	Yan'an, Shaanxi	1
	Brassica napus L.	2022. 04	Jinhua, Zhejiang	2
Apis cerana honey (ACH)	Polyflora	2017. 11	Shennongjia Forestry District, Hubei	2
	Litchi chinensis Sonn.	2018. 04	Shenzhen, Guangdong	2
	Brassica napus L.	2018. 04	Xinghua, Jiangsu	1
	Brassica napus L.	2020. 03	Quzhou, Zhejiang	2
	Ziziphus jujuba Mill.	2020. 06	Yan'an, Shaanxi	2
	Rhus chinensis Mill.	2020. 06	Yan'an, Shaanxi	2
	Polyflora	2020. 11	Shennongjia Forestry District, Hubei	3
	Eurya japonica Thunberg	2021. 03	Shangrao, Jiangxi	1
	Polyflora	2021. 04	Wenzhou, Zhejiang	1
	Leucosceptrum canum Smith	2021. 05	Dehong, Yunnan	2
	Polyflora	2021. 11	Enshi, Hubei	2
	Eriobotrya japonica Lindl.	2021. 12	Hangzhou, Zhejiang	1
	Polyflora	2022. 03	Hangzhou, Zhejiang	1
	Polyflora	2022. 05	Chongzuo, Guangxi	2
Apis laboriosa honey (ALH)	Polyflora	2020. 04	Honghe, Yunnan	3
	Polyflora	2020. 05	Pu'er, Yunnan	1
	Polyflora	2020. 05	Lincang, Yunnan	3
	Polyflora	2020. 05	Tibet	1
	Polyflora	2021. 04	Xishuangbanna, Yunnan	1
	Polyflora	2021. 05	Dehong, Yunnan	3
	Polyflora	2022. 04	Dehong, Yunnan	3
	Polyflora	2022. 04	Xishuangbanna, Yunnan	3
	Polyflora	2020. 05	Hainan	2
	Polyflora	2020. 06	Xishuangbanna, Yunnan	4
	Polyflora	2020. 06	Guangxi	1

Apis dorsata honey (ADH)	Polyflora	2021. 04	Xishuangbanna, Yunnan	4
	Polyflora	2021. 06	Xishuangbanna, Yunnan	3
	Polyflora	2022. 04	Xishuangbanna, Yunnan	4
	Polyflora	2022. 05	Chongzuo, Guangxi	1
	Polyflora	2022. 05	Baise, Guangxi	1
Apis florea honey (AFH)	Polyflora	2020. 06	Xishuangbanna, Yunnan	2
	Polyflora	2020. 07	Honghe, Yunnan	2
	Polyflora	2021. 04	Xishuangbanna, Yunnan	4
	Polyflora	2021. 06	Xishuangbanna, Yunnan	4
	Polyflora	2022. 04	Chongzuo, Guangxi	2
	Polyflora	2022. 05	Chongzuo, Guangxi	3
	Polyflora	2022. 05	Yuxi, Yunnan	2
	Polyflora	2022. 06	Honghe, Yunnan	2
	Polyflora	2022. 09	Yuxi, Yunnan	1
	Polyflora	2022. 09	Honghe, Yunnan	3

Table S3. Sequencing data of microbial community in honey samples

	Raw reads	Effective reads	ASVs	Species count at different taxonomic levels				
				Phylum	Class	Order	Family	Genus
AMH1	102520	97726	194	9	10	31	58	89
AMH2	119190	113750	428	20	35	74	124	191
AMH3	103382	97862	300	19	29	57	95	138
ACH1	105927	100849	570	21	39	85	131	219
ACH2	110824	106326	482	20	35	77	120	183
ACH3	102617	98660	486	17	30	69	118	178
ALH1	102403	97710	377	17	21	50	84	111
ALH2	103649	98875	418	16	25	58	91	125
ALH3	104780	101385	319	12	20	50	70	107
ADH1	118171	112448	707	22	31	74	127	205
ADH2	55877	53467	507	18	28	69	107	174
ADH3	102468	98825	677	20	30	71	120	200
AFH1	106459	101860	364	12	19	45	77	128
AFH2	103541	96614	191	12	16	35	60	86
AFH3	81719	79392	286	11	13	38	64	95

ASVs: Amplicon sequence variants

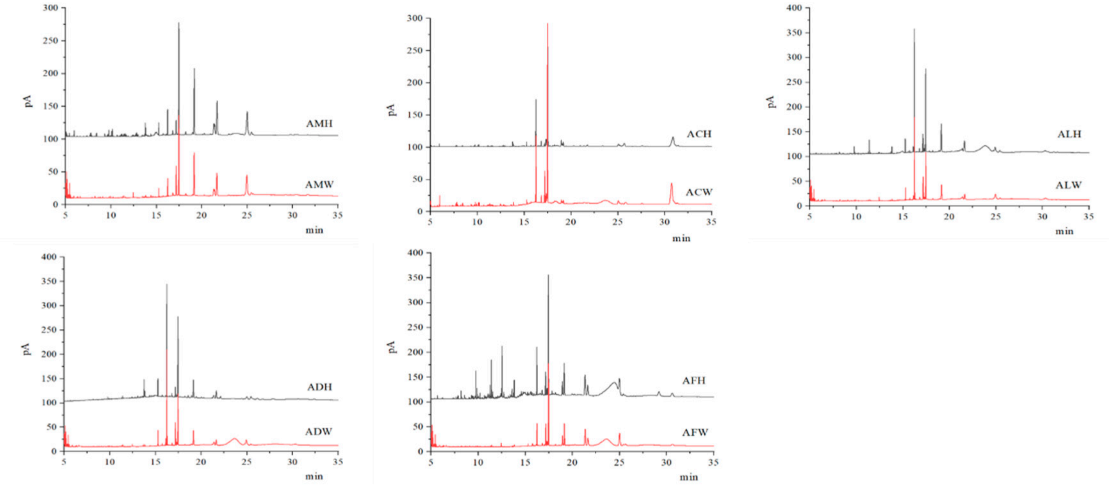

Figure S1. Gas chromatography of petroleum ether extracts from honey and beeswax of different honey bee species

AMH: *A. mellifera* honey; ACH: *A. cerana* honey; ALH: *A. laboriosa* honey; ADH: *A. dorsata* honey; AFH: *A. florea* honey; AMW: *A. mellifera* beeswax; ACW: *A. cerana* beeswax; ALW: *A. laboriosa* beeswax; ADW: *A. dorsata* beeswax; AFW: *A. florea* beeswax
